# *BRAF* V600K vs. *BRAF* V600E: a comparison of clinical and dermoscopic characteristics and response to immunotherapies and targeted therapies

**DOI:** 10.1111/ced.15113

**Published:** 2022-03-11

**Authors:** Corrado Zengarini, Martina Mussi, Giulia Veronesi, Aurora Alessandrini, Martina Lambertini, Emi Dika

**Affiliations:** ^1^ Division of Dermatology IRCCS Azienda Ospedaliero‐Universitaria di Bologna Bologna Italy; ^2^ Division of Dermatology, Department of Experimental, Diagnostic and Specialty Medicine University of Bologna Bologna Italy

## Abstract

**Background:**

A number of mutations related to malignant melanoma (MM) have been identified, and of the mutated genes, *BRAF* has been found to be altered in > 50% of cases. Most of these have been *BRAF* V600E mutations, whereas the incidence of *BRAF* V600K may vary from 10% to 30%. Little is known about the clinical prognostic correlations of *BRAF* V600K MMs. We evaluated the clinical and dermoscopic features, incidence, therapy response and outcomes in the medium to long term.

**Aim:**

To compare the clinical and dermoscopic characteristics, the response to systemic therapies and the prognosis among MMs with *BRAF* V600E and *BRAF* V600K mutations.

**Methods:**

We retrieved the data of patients tested in our centre for MM from 2012 to 2015, including clinical features, dermoscopic pictures, clinical history and tumour mutations. Only patients with *BRAF* V600E and *BRAF* V600K mutations were included. Any MMs positive for *BRAF* V600K mutation were collected, and the number of V600K cases and their features were used to extract the same number of patients with *BRAF* V600E from our database using a matching method. The clinical and dermoscopic presentation, therapy response and disease progression of the two groups were then evaluated.

**Results:**

In total, 132 cases of *BRAF* V600E‐mutated MMs were identified, and then randomized with a propensity‐score method to match the 10 retrieved cases of *BRAF* V600K mutation. Both groups had a nodular appearance to the tumours and an advanced disease stage, and no significant differences in dermoscopic features were highlighted. During the follow‐up period, four patients with *BRAF* V600K died of disease‐specific causes. Moreover, we found a higher frequency of metastasis, a faster disease progression and more rapid mortality in patients with *BRAF* V600K.

**Conclusion:**

Despite the small size of this study, the results show similar clinical and dermoscopic characteristics between V600E and V600K mutations, but compared with *BRAF* V600E MMs, *BRAF* V600K MMs seem to be less responsive to therapy and have a worse prognosis.

## Graphical abstract

Our work shows that melanomas affected by the *BRAF* V600K variant have a similar clinical and dermoscopic presentation but a more aggressive behaviour than those with *BRAF* V600E.

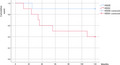

## Introduction

Malignant melanoma (MM) is a neoplasm that results from uncontrolled proliferation of melanocyte cells.^1^ Although it is only the third most common cutaneous neoplasia, it has the highest mortality rate of all cutaneous malignancies.

Uncontrolled activation of the mitogen activated protein kinase (MAPK) signalling pathway is principally involved in MM pathogenesis.^2^ In particular, this pathway can induce cell growth, proliferation and differentiation while inhibiting apoptosis by means of growth factors. Several key genes are involved in this pathway, including *RAS*, *BRAF*, *MEK* and *ERK*.^3^ More than 90% of cutaneous MMs present constitutive MAPK pathway activity. In particular, the prevalence of mutations in codon 600 of *BRAF* ranges between 40% and 60% in patients with MM. The most prevalent mutations in MM are the *BRAF* V600E variant (about 80%) and *BRAF* V600K (5–30%), with other subtypes found at lower frequencies: V600M 4%, V600R 5% and V600D < 5%.^4^

Heterogeneity in *BRAF* mutations within the same patient has been described, with differences between primary tumours and metastases, and between different metastases.^5^ Testing for *BRAF* mutation status is usually performed on the most recently resected or biopsied tumours. It is important to note that although *BRAF* inhibitors show comparative efficacy in both the V600E and V600K mutations, the two tumour types have sometimes been considered as distinct entities.^6^ Indeed, recent scientific studies reported that *BRAF* V600K MMs, in contrast to *BRAF* V600E MMs, seem to increase with age and have a higher risk for brain and lung metastases and a shorter time from diagnosis to metastasis onset and patient death.^7^ Several reports of patients with *BRAF* V600K‐mutated MMs also described significant differences in sex, age, primary MM location, the interval from MM first diagnosis to Stage IV disease and the overall survival after the diagnosis of Stage IV disease. In particular, the V600K mutation was significantly associated with older age, male sex, head and neck as the primary MM site, short overall survival from the time of diagnosis of Stage IV disease and a higher degree of chronic sun damage, which might explain the variable geographical frequency of *BRAF* V600K.^8^

Finally, although Pozzobon *et al*.^9^ previously analysed the dermoscopic criteria associated with *BRAF* and *NRAS* mutation status in primary cutaneous MM, very little is known about the dermoscopic and prognostic differences between V600E and V600K variants and the correlation of dermoscopic features and mutational burden.^9^, ^10^, ^11^ We therefore performed this study to compare the clinical and dermoscopic characteristics of MMs with these two mutations and the response to immunotherapies and targeted therapies.

## Methods

The study was approved by the ethical committee at our institution (no. DERM‐MTC 2017). No informed consent was required.

### Study design

We conducted a retrospective analysis of patients registered at our Melanoma Unit at the Policlinico Sant'Orsola‐Malpighi, Bologna, Italy. The data are available on request from the authors.

### Patient collection

We built a database using data collected by primary search from our internal register of anatomopathological reports, collecting all the cases of MMs to analyse for *BRAF* mutations. Only patients with at least 5 years of follow‐up during the period 2012–2015 and treated with immune or targeted therapies were included.

We used next‐generation sequencing technique for mutation analysis, using a multigenic panel (Oncomine Focus Assay; ThermoFisher Scientific, Waltham, MA, USA).^12^

Patients considered as having been treated with targeted and immunotherapies only if a cycle of treatment, consisting of the standard dosage and with a duration of at least 12 months, had been performed with at least three control computed tomography scans for evaluation.

Independent variables such as sex, age, tumour location on the skin, treatment type, TNM stage and Breslow thickness were registered in this purpose‐built database. Body sites for tumour location was included the limbs, trunk, face, scalp, back, hands, feet and anogenital area.

### Melanoma collection

All MMs positive for *BRAF* V600K mutation were collected. The number of V600K cases and their features were used to extract the same number of patients with *BRAF* V600E from the database using a matched propensity score method. Matching criteria including patient sex and age, Breslow thickness, tumour site, TNM and therapies in use. Dermoscopic images of cases belonging to both groups were downloaded from our videodermoscopy database.

### Comparison of the groups

We compared the two groups for the characteristics of interest. The clinical presentation of the tumour was assessed as one of three patterns: nodular, ulcerated nodular and superficial.

The evaluated dermoscopic characteristics included vascular pattern, blue–white veil, chrysalids, blue–grey blotches, reticular grey–blue areas, white regression structures, peppering, and presence of > 3 colours. Three trained dermoscopists have evaluated magnified images registered in our database. Each dermoscopic criteria have been singularly evaluated and each dermatologist was blinded to patient identity, clinical information and mutational status at the time of the assessment.

Response to therapy was evaluated by analysing the trend of metastasis numbers and volume on three consecutive CT scans after therapy administration. Outcomes considered were no metastasis; tumour in remission, stable, with partial improvement or no improvement; mortality rate; time to metastasis onset (months); and time to death (months).

### Statistical analysis

The *BRAF* V600E group was extracted from a larger group using a matched propensity score method. Approximation values for independent variables with a tolerance of 20% were used to reach 1 : 1 ratios in both groups. The two groups were then rechecked for homogeneity using the Mann–Whitney *U*‐test for quantitative variables and Fisher exact test for qualitative variables. Quantitative data were then expressed as mean ± SD and an independent samples Mann–Whitney *U*‐test was used to compare the two groups. Qualitative data were described using the Fisher exact probability method. *P* < 0.05 was considered statistically significant. Kaplan–Meier curves were used to represent the mortality and metastasis onset between the two groups of patients. Data processing and statistical analysis were performed using an Excel (Microsoft Corp, Redmond, WA, USA) database and SPSS software (V26; IBM SPSS, Armonk, NY, USA).

## Results

### Patients

From the search in our hospital database, we found 223 patients positive for *BRAF* V600 mutations and with at least 5 years of follow‐up. Of these, 132 patients were positive for the *BRAF* V600E variant and 10 patients for the *BRAF* V600K mutation. Matching these groups, we identified 10 patients whose independent variables were not statistically different from that of patients with *BRAF* V600K.

### Characteristics

The mean age was 57.7 years for the *BRAF* V600E group, and 65.90 years for the *BRAF* V600K groups (Table 1). There was a male predominance in both groups, with a male/female ratio of 7 : 3 for *BRAF* V600E and 8 : 2 for *BRAF* V600K.

**Table 1:** Demographic characteristics, tumour thickness at diagnosis^a^ and systemic therapies of the two groups.

Parameter	*BRAF* mutation		*P*
	V600E	V600K	
Age, years^b^	57.70 ± 13.458	65.90 ± 3.139	0.14^c^
Sex M : F	7 : 3	8 : 2	1.00^d^
Breslow thickness, mm^b^	2.35 ± 10.52	2.73 ± 11.26	0.48
Systemic therapies	70% dabrafenib plus trametinib; 10% nivolumab; 10% ipilimumab; 10% NS	70% dabrafenib plus trametinib; 10% nivolumab; 10% vemurafenib plus cobimetinib; 10% NS	1.00

NS, not stated.

^a^ There was no difference between the groups in tumour site (arms, lower legs, thighs, feet, hands, face, scalp, trunk, genital area) or TNM stage (each TNM was assigned with a numeric number, e.g. IA = 1, IB = 2, etc.).

^b^ Mean ± SD.

^c^ Mann–Whitney *U*‐test.

^d^ Fisher exact test.

The macroscopic clinical analysis did not show any significant differences (Table 2). Both groups had the same frequency of affected areas (arms, legs, thighs, feet, hands, face, scalp, trunk and genitals).

**Table 2:** Comparison of the clinical presentation, dermoscopic features response to therapies and outcomes of the two groups.

Parameter	*BRAF* mutation		*P*
	V600E	V600K	
Clinical presentation, *n*			1.00
Nodular	3	3	
Ulcerated nodular	5	4	
Superficial	2	3	
Dermoscopy findings, *n* ^a^			
Vessels	4	3	0.31
Blue–white veil	3	4	0.61
Chrysalids	3	2	0.53
Peppering	1	2	0.44
> 3 colours	4	5	0.44
Blue–grey blotches	4	4	0.59
Reticular grey–blue areas	3	3	0.66
White regression	5	4	0.66
Immune therapy response, *n*			0.48^b^
Not evaluable	1	2	
None	0	5	
Partial	1	1	
Stable	8	2	
Reduction	0	0	
Metastasis, *n*			
Yes	5	9	0.07
No	5	1	
Died, *n*			
Yes	1	4	0.03
No	9	6	

^a^ Seven images of *BRAF* V600E (3) and *BRAF* V600K (4) were not found and thus could not be evaluated.

^b^ Mann–Whitney *U*‐test; all other *P* values were assessed using Fisher test.

The mean Breslow thickness was 2.35 mm for *BRAF* V600E and 2.73 mm for *BRAF* V600K. Based on the American Joint Committee on Cancer guidelines (eighth edition) there was no difference in TNM stage evaluated between the two groups for the same Breslow thickness.

### Comparison of the two groups

The tumours were classified in accordance with their appearance, with ulcerated nodular being the most common, followed by nodular and then superficial.

There was lower statistical power for the dermoscopic feature due to the lack of three images in the *BRAF* V600K group and four images in the *BRAF* V600E group, making a total loss of seven cases (35%). However, analysis of the remaining samples did not demonstrate any noteworthy characteristics for either group.

In the *BRAF* V600E group, 70% of the cases were treated with a combination of dabrafenib and trametinib, while 10% were treated with nivolumab and 10% with ipilimumab; for the remaining 10% of patients, it was not possible to identify the therapy because of incomplete medical records. The *BRAF* V600K groups also had 70% treated with a combination of dabrafenib and trametinib, while 10% were treated with nivolumab and 10% with a combination of vemurafenib and cobimetinib; again, it was not possible to identify the therapy for the remaining 10% of patients because of incomplete medical records.

The study identified an interesting difference in the response to therapies, with a better outcome achieved in the *BRAF* V600E group; eight patients in this group had stable response, compared with partial or no response in six patients in the *BRAF* V600K group.

**Figure 1:**
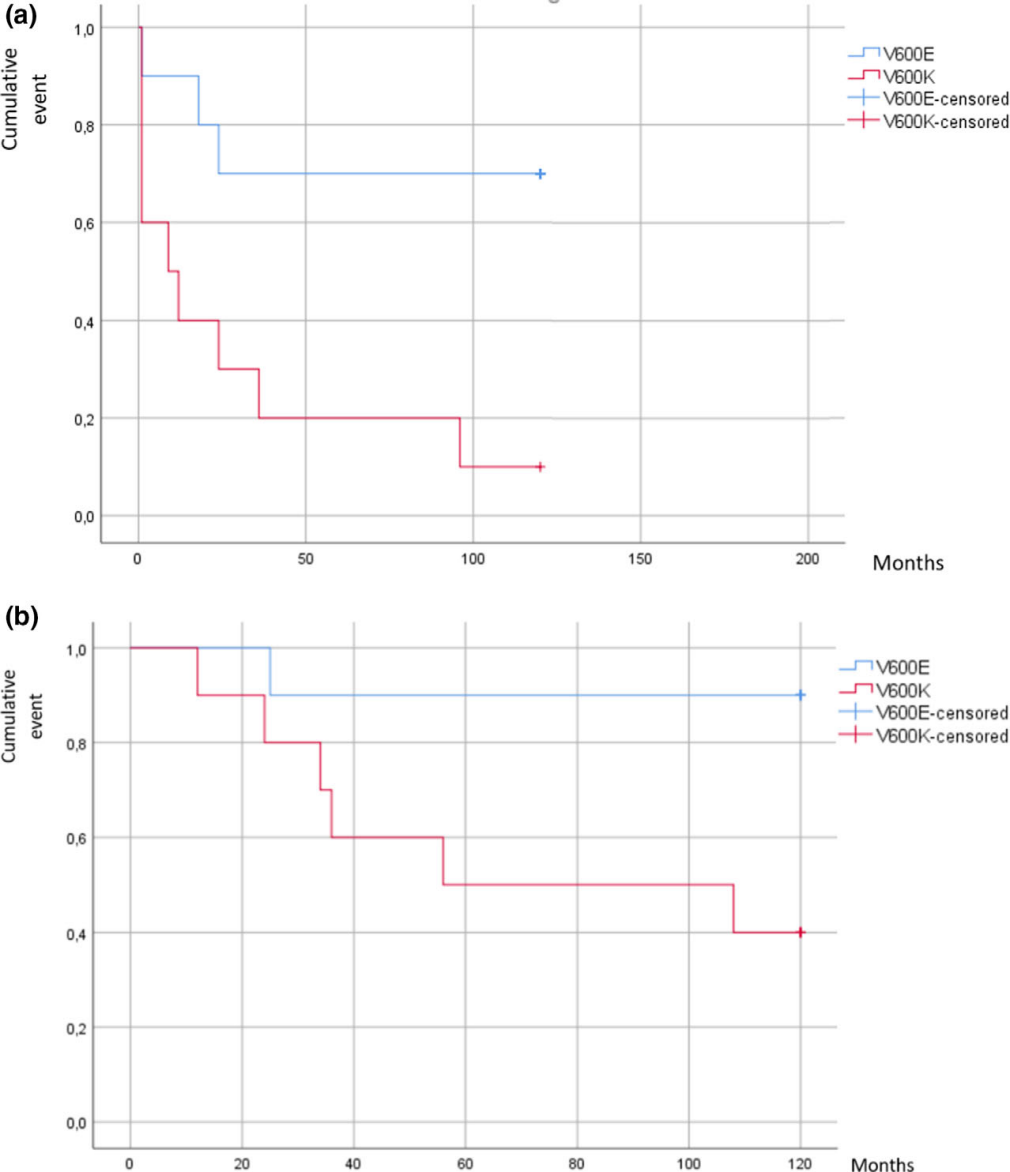
(a,b) Kaplan–Meier plots of (a) development of metastases diagnosed by computed tomography scan in the two groups, showing the faster progression in *BRAF* V600K cancers; and (b) disease‐specific mortality, showing that half of the patients with *BRAF* V600K died within 40 months. [Colour figure can be viewed at wileyonlinelibrary.com]

During the follow‐up period, four patients with *BRAF* V600E and nine patients with *BRAF* V600K died (Fig. 1a), which was statistically significant (*P* < 0.03). Finally, a similar but not statistically significant outcome was observed in metastasis onset, which developed more rapidly in the *BRAF* V600K group than in the *BRAF* V600E group (Fig. 1b).

## Discussion

Specific mutations in MMs lead to differences in prognosis, therapy administration and response for wild‐type vs. *BRAF*‐mutated tumours. We know that *BRAF*‐mutated MMs are linked to a worse prognosis.^13^ However, there is still controversy as to how different mutations of the same gene locus can change cancer prognosis, especially with regard to *BRAF* variants. Of the various *BRAF* mutations, the best‐known and most frequent variants are the V600E and V600K codon changes. Some studies have already explored the differences, clinical and histopathological characteristics, and predictive behaviour of these two mutation types, and have shown that *BRAF* V600K mutations appear to be more aggressive than the *BRAF* V600E mutations,^14^ and our results are in agreement with these.

Despite the small size of our study, comparison of the two groups with the previous propensity matching technique^15^ reduced any variability due to the independent parameters that could have influenced the results and could not have been excluded in later analysis. Once we established that the two groups did not show significant differences in characteristics and had undergone very similar therapeutic protocols, we were able to highlight the differences between them, excluding the most apparent confounding factors.

We analysed whether there was a difference in risk of metastases, micrometastases and sentinel lymph node involvement in patients with tumours of the same Breslow thickness, which would indicate more significant initial aggression. The analysis showed no distinct differences, suggesting that both mutations have a similar initial TNM stage.

Regarding the type of clinical presentation at macroscopic level, both groups had the same frequency of tumour types, namely superficial (flat) nodular or ulcerated appearance.

Because of the missing dermoscopic images, our sample appears too small for analysis to confirm a significant pattern between the two groups; however, the analysis of the remaining cases did not highlight any specific differences between the groups. Furthermore, also white regression and peppering has been reported by other groups, we did not find such a high prevalence of this feature in our study, casting doubt on its correlation with *BRAF* V600 mutations.^9^

By contrast, the analysis of the response to systemic treatments was clearer; in the *BRAF* V600E group, although it was challenging to achieve remission of metastases, 8 of the 10 patients had at least a stable response, whereas in the *BRAF* V600K group, 6 of the 10 patients achieved little or no response. These results strongly suggest stronger resistance to targeted therapies by *BRAF* V600K‐mutated tumours compared with the more widespread *BRAF* V600E variant, which agrees with previous studies.^16^ In addition, we observed more rapid spread of metastasis in the *BRAF* V600K, with 50% of the patients developing metastases in the first year after the first diagnosis, indicating a more aggressive course. The observed mortality rate was in accordance with the metastasis rate, with higher mortality from disease‐related causes occurring in the *BRAF* V600K compared with the *BRAF* V600E group, with a ratio of 4 : 1, respectively, and approximately half of the patients in the *BRAF* V600K group died within 3 years of diagnosis.

## Conclusion

Despite the small size of our study being a limitation the results allowed us to identify some interesting elements and distinctive traits between the two groups. There were no differences in clinical features or dermoscopic patterns between the two variants. We could not identify any clinical feature that, without molecular analysis, could lead to one mutation being suspected over the other.

However, we did find differences in therapy response, metastasis development and mortality rate, with *BRAF* V600K‐mutated MMs showing lower response rate to therapies and a more rapid and frequent tendency to develop metastasis, implying greater resistance to the proposed treatments. Consequently, patients with *BRAF* V600K‐mutated MMs also had a more rapid and increased mortality rate during follow‐up.

In summary, this work shows that MMs with the *BRAF* V600K mutation have a similar clinical presentation but more aggressive behaviour than those with the *BRAF* V600E mutation.

**What's already known about this topic?**

• *BRAF* V600K MMs have different clinical and dermoscopic features, a higher risk for developing metastases and a worse prognosis compared with *BRAF* V600E MMs.
• *BRAF* V600K is more resistant to systemic therapies in comparison to *BRAF* V600E.

**What does this study add?**

• We found nonsignificant differences in the clinical and dermoscopic presentations of the two principal mutations.
• *BRAF* V600K tumours showed significantly greater resistance to systemic therapies and increased development and progression of metastases and mortality.
